# Biologic therapy for Crohn’s disease (2015–2025): a bibliometric analysis of global research trends and current status

**DOI:** 10.3389/fphar.2026.1943640

**Published:** 2026-09-22

**Authors:** Pengfei Zhou, Hui Pan, Manxiang Wei, Yanyang Zhou, Haitao Qu, Jiawen Wang, Youcheng He

**Affiliations:** 1 Anorectal Department, The First Affiliated Hospital of Henan University of CM, Zhengzhou, Henan, China; 2 Coloproctology Department, Longhua Hospital, Shanghai University of Traditional Chinese Medicine, Shanghai, China; 3 Department of Gastroenterology, Second People’s Hospital Affiliated to Fujian University of Traditional Chinese Medicine, Fuzhou, Fujian, China

**Keywords:** bibliometric analysis, biologic therapy, Crohn’s disease, inflammatory bowel disease, precision medicine

## Abstract

**Background:**

Crohn’s disease (CD) is a chronic inflammatory bowel disease with rising global prevalence. Biologic agents have become the cornerstone of treatment for moderate-to-severe CD, yet the rapidly expanding literature makes systematic tracking of research trends challenging. This study presents a comprehensive bibliometric analysis to systematically map the research landscape of biologic therapy for CD from 2015 to 2025.

**Methods:**

Publications on biologic therapy for CD were retrieved from the PubMed, Web of Science Core Collection, and Scopus databases for the period from 1 January 2015 to 31 December 2025. To ensure consistency in interpretation and indexing across databases, only English-language articles were included. After deduplication using EndNote and manual screening, 15,828 eligible publications were included in the final analysis. Bibliometric and visualization analyses were performed using CiteSpace, VOSviewer, RStudio, and SCImago Graphica. Metrics including annual publication output, country contributions with centrality analysis (indicating a country’s bridging role in international collaboration networks), institutional collaboration networks, author co-authorship patterns, keyword co-occurrence, cluster analysis, timeline visualization, and citation bursts were systematically examined.

**Results:**

The annual publication output showed a consistent upward trend. The United States (n = 4,505), China (n = 1,528), and Italy (n = 1,509) were the top three producers; Canada showed the highest centrality (0.93), reflecting its role as a key bridge in transnational collaborations. Keyword co-occurrence revealed a trilevel structure centered on CD, with “biological marker” and “biological product” appearing at near-equal frequencies. Eleven clusters were identified (Q = 0.5991, silhouette = 0.8053), and 25 citation bursts revealed a three-phase evolution from efficacy validation to strategy optimization.

**Conclusion:**

The bibliometric findings demonstrate that CD biologic therapy research has established a mature ecosystem, with shifting emphases toward biomarker-driven stratification and comparative effectiveness. Future priorities emerging from the data include individualized sequencing strategies, head-to-head comparisons with novel small-molecule agents, and expanded investigation in understudied populations such as adolescents and pregnant women.

## 1 Introduction

Crohn’s disease (CD) is a chronic, relapsing, and progressive inflammatory disorder of the intestinal tract that can affect any segment of the digestive system. It is frequently complicated by strictures, fistulas, and perianal lesions, which substantially impair patients’ quality of life. Approximately 40%–50% of patients require surgical intervention within 10 years of diagnosis (Burisch et al., 2019). The pathogenesis of CD is multifactorial, involving genetic susceptibility, immune dysregulation, gut microbiota alterations, and environmental factors. Conventional pharmacotherapy includes corticosteroids, 5-aminosalicylates, and immunomodulators, while nutritional therapy also plays an important role in specific populations such as pediatric patients and those with mild-to-moderate disease.

In recent years, biologic agents have assumed a pivotal role in the treatment of moderate-to-severe CD. Biologics are macromolecular drugs—including monoclonal antibodies and antibody-fusion proteins—produced through modern biotechnology, which exert precise actions on specific targets within the inflammatory cascade. The first tumor necrosis factor (TNF) inhibitor, infliximab, has been in clinical use for over 25 years (Nielsen and Ainsworth, 2013). Subsequently, agents targeting immune cell trafficking (vedolizumab) or cytokine signaling (ustekinumab and risankizumab) have been developed and widely adopted (Sandborn et al., 2013; MacDonald et al., 2016). The therapeutic landscape has also been reshaped by the introduction of biosimilars and, more recently, small-molecule agents such as JAK inhibitors, though the latter are not the focus of this analysis.

The period from 2015 to 2025 represents a critical period for biologic therapy in CD. This timeframe captures the maturation of the field following the establishment of anti-TNF agents as standard care, the widespread introduction of newer biologics with distinct mechanisms (vedolizumab, ustekinumab, and risankizumab), the market entry of biosimilars, and the growing emphasis on treat-to-target strategies and biomarker-driven precision medicine. Analyzing this specific window allows us to capture the transition from efficacy validation to therapeutic optimization—a shift that defines the contemporary research agenda.

Given the rapid expansion and increasing complexity of the literature, a systematic quantitative assessment is warranted. In this study, we conducted a comprehensive bibliometric analysis of publications on biologic therapy for CD from 2015 to 2025, using data retrieved from PubMed, Web of Science Core Collection, and Scopus. The specific objectives were: (1) to characterize annual publication trends and geographic distribution; (2) to identify productive countries, institutions, and authors, and map their collaboration networks; (3) to delineate core research themes, temporal evolution, and emerging hotspots through keyword co-occurrence, cluster analysis, and citation burst detection; and (4) to synthesize these findings to inform future research directions. By systematically mapping the intellectual structure of this field, this analysis aims to provide researchers, clinicians, and policymakers with an evidence-based overview of the current state and evolving priorities in biologic therapy for CD.

## 2 Methods

### 2.1 Data source and search strategy

We searched the PubMed, Web of Science Core Collection, and Scopus databases using “Crohn’s disease” AND “biologicals” to identify all relevant publications published between 1 January 2015 and 31 December 2025. Search method: PubMed: (“Crohn Disease” [MeSH] OR “Crohn’s disease” [Title/Abstract] OR “Crohn disease” [Title/Abstract]) AND (“Biological Products” [MeSH] OR “Biological Products” [Title/Abstract] OR “Biologics” [Title/Abstract] OR “Biological Therapy” [MeSH] OR “Biological Therapy” [Title/Abstract]. Web of Science Core Collection: TS= (“Crohn’s disease” OR “Crohn disease”) AND TS= (biologic OR biological OR biological products OR biological agents). Scopus: TITLE-ABS-KEY (“Crohn’s disease” OR “Crohn disease”) AND TITLE-ABS-KEY (biologic OR biological OR biological products OR biological agents). The final search was performed on 30 January 2026. The search was restricted to articles written in English. Title and abstract screening were performed independently by two reviewers (Pengfei Zhou and Hui Pan). Studies were excluded sequentially: first by title, then by abstract, based on eligibility criteria concerning study population, intervention, and study design. Full texts of potentially eligible records were retrieved for final assessment. Disagreements were settled by discussion or by adjudication of a third reviewer (Youcheng He). The screening process is summarized in a PRISMA flowchart (Figure 1A), with exclusion details reported in the Results section.

**FIGURE 1:**
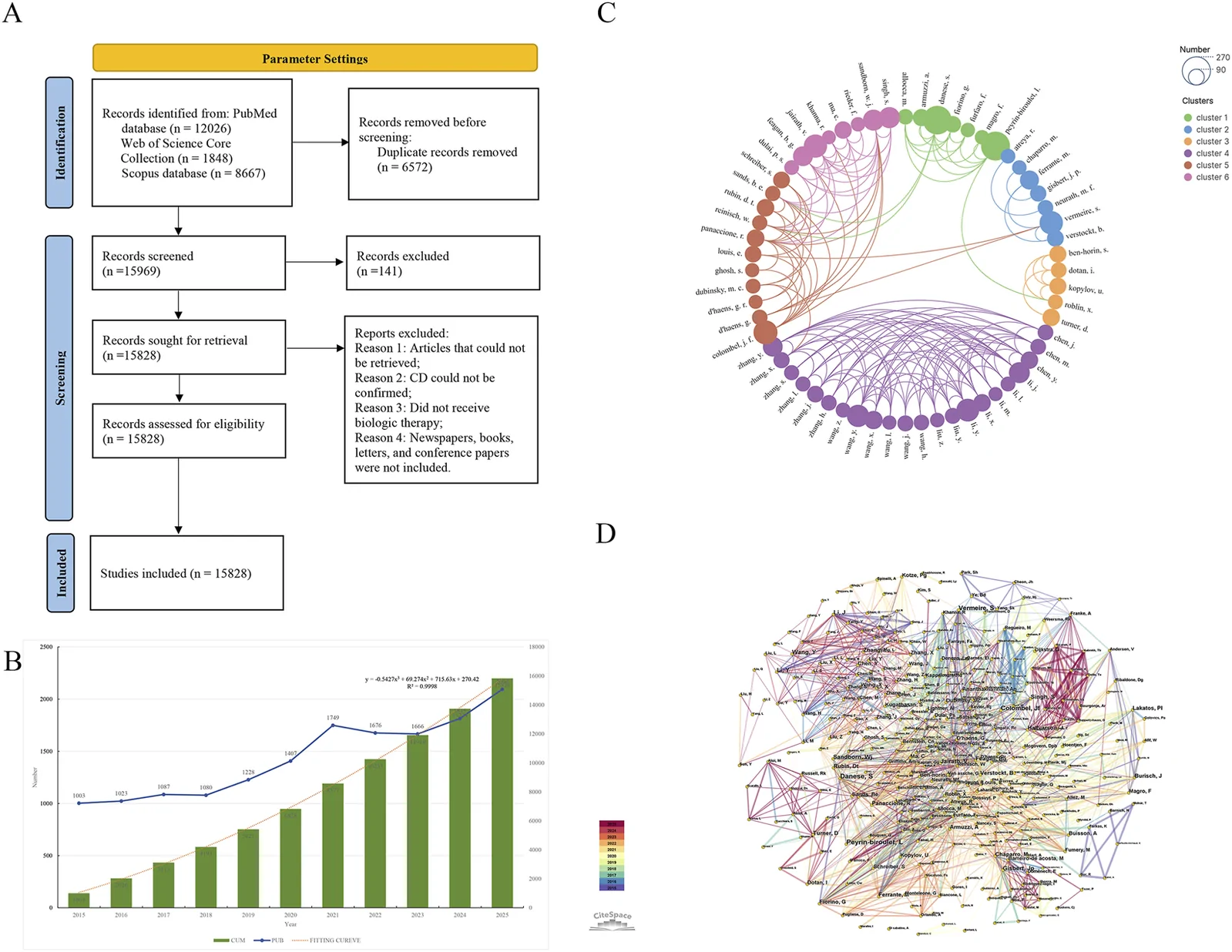
Literature search, publication distribution, and author collaboration network. **(A)** Flow diagram of the literature search and screening process (PRISMA Flowchart). **(B)** Annual distribution of publications on biologic therapy for Crohn’s disease from 2015 to 2025. **(C)** Ring diagram of author collaborations. **(D)** Co-authorship network visualization of the leading authors.

### 2.2 Inclusion and Exclusion Criteria

Inclusion Criteria: 1. Publication type was original research (including randomized controlled trials, cohort studies, case-control studies, and case series), meta-analyses, animal experimentation, or systematic and reviews; 2. Crohn’s disease patients or animal models; 3. Received treatment with at least one biological agent; 4. The publication date fell between 1 January 2015 and 31 December 2025; 5. The article was written in English.

Exclusion Criteria: 1. Articles that could not be retrieved; 2. CD could not be confirmed; 3. Did not receive biologic therapy; 4. Newspapers, books, letters, and conference papers were not included.

### 2.3 Software and analytical methods

Literature deduplication and merging were performed using EndNote 2025 (Bid19000). Duplicate detection was performed using EndNote’s ‘Find Duplicates’ function based on title, author, year, and journal, followed by manual verification of ambiguous cases. Bibliometric analysis and visualization were conducted using CiteSpace 7.0. R0, R version 4.5.1 with RStudio 2025.05.1 + 513, SCImago Graphica Beta 1.0.55, and VOSviewer version 1.6.20. For CiteSpace analysis, time slicing: January 2015 to December 2025, with 1-year increments; Node types: keyword, country, institution, author; Selection criteria: Top N = 50 per time slice, We selected Top N = 50 per time slice as this threshold is standard in bibliometric studies, providing a balance between computational feasibility and sufficient node inclusion to capture the core research structure without excessive noise; Pruning: pathfinder and pruning sliced networks; Visualization: cluster view with timeline view. The modularity Q value (threshold >0.3) and silhouette value (threshold >0.7) were used to evaluate the quality of clustering, indicating statistically significant and internally homogeneous clusters, respectively. Centrality values (>0.1) were interpreted as indicating nodes serving as important bridges within the collaboration network, following standard bibliometric convention.Geographic visualization of publication output by country and figure compilation were performed using the “ggplot2”, “dplyr”, and “cowplot” packages in RStudio. Publication trend graphs were generated using Microsoft Office Excel 2019. Keyword density visualization was generated using VOSviewer, and the circular collaboration network of authors was generated using SCImago Graphica in conjunction with VOSviewer.

## 3 Results

### 3.1 Literature screening

A systematic literature search was performed across three databases, yielding a total of 12,026 records from PubMed, 1,848 records from the Web of Science Core Collection, and 8,667 records from Scopus. All retrieved records were then imported into EndNote 2025 for deduplication. After removing 6,572 duplicate records, 15,969 unique records remained. During title/abstract screening and full-text assessment, 141 records were excluded for the following reasons: not related to Crohn’s disease; not involving biologic therapy; non-original or non-review article types such as conference abstracts or editorials; full text not retrievable; duplicate publications not captured by automated deduplication; studies not reporting original data or not meeting the predefined intervention criteria. Ultimately, 15,828 eligible publications were included in the final bibliometric analysis (Figure 1A).

### 3.2 General publication trends

Bibliometric analysis of the included publications from 2015 to 2025 demonstrated a consistent upward trend in research output on biologic therapy for Crohn’s disease (Figure 1B).

### 3.3 Cooperation with the author

A total of 410 authors were identified as having participated in the published research on biologic therapy for Crohn’s disease during the 2015–2025 period. The 410 authors represent those with sufficient publication activity for meaningful network analysis. Among them, the 10 most productive authors, based on the number of publications, were Peyrin-Biroulet, L (282 publications), Danese, S (274), Colombel, JF (204), Vermeire, S (188), Jairath, V (170), Li, Y (154), Singh, S (146), Sandborn, WJ (142), Wang, Y (139), and Li, J (134). The collaboration network and co-authorship patterns among these leading authors are visualized in Figures 1C,D.

### 3.4 Country of publication

The top 10 countries in terms of publication output were the United States (n = 4,505), China (n = 1,528), Italy (n = 1,509), Canada (n = 1,284), the United Kingdom (n = 1,016), Germany (n = 987), France (n = 969), Spain (n = 916), Japan (n = 626), and Belgium (n = 593). The United States ranked first in publication volume and had the most extensive international collaboration network in terms of total collaborative links. However, a country’s centrality value reflects its role as an intermediary that connects different research clusters, rather than the total volume of its collaborations. Canada, with a centrality value of 0.93, emerged as a particularly critical intermediary node in the transnational collaboration network. These observations indicate that publication productivity does not necessarily align with network connectivity efficiency, and underscore the need for further exploration of the structural dynamics underlying international research cooperation. (Table 1; Figure 2A).

**TABLE 1:** Top 10 countries in terms of number of publications.

Rank	Country	Volume of publications	Centrality
1	United States	4505	0.13
2	China	1528	0.87
3	Italy	1509	0.15
4	Canada	1284	0.93
5	United Kingdom	1016	0.15
6	Germany	987	0.29
7	France	969	0.38
8	Spain	916	0.59
9	Japan	626	0.10
10	Belgium	593	0.87

**FIGURE 2:**
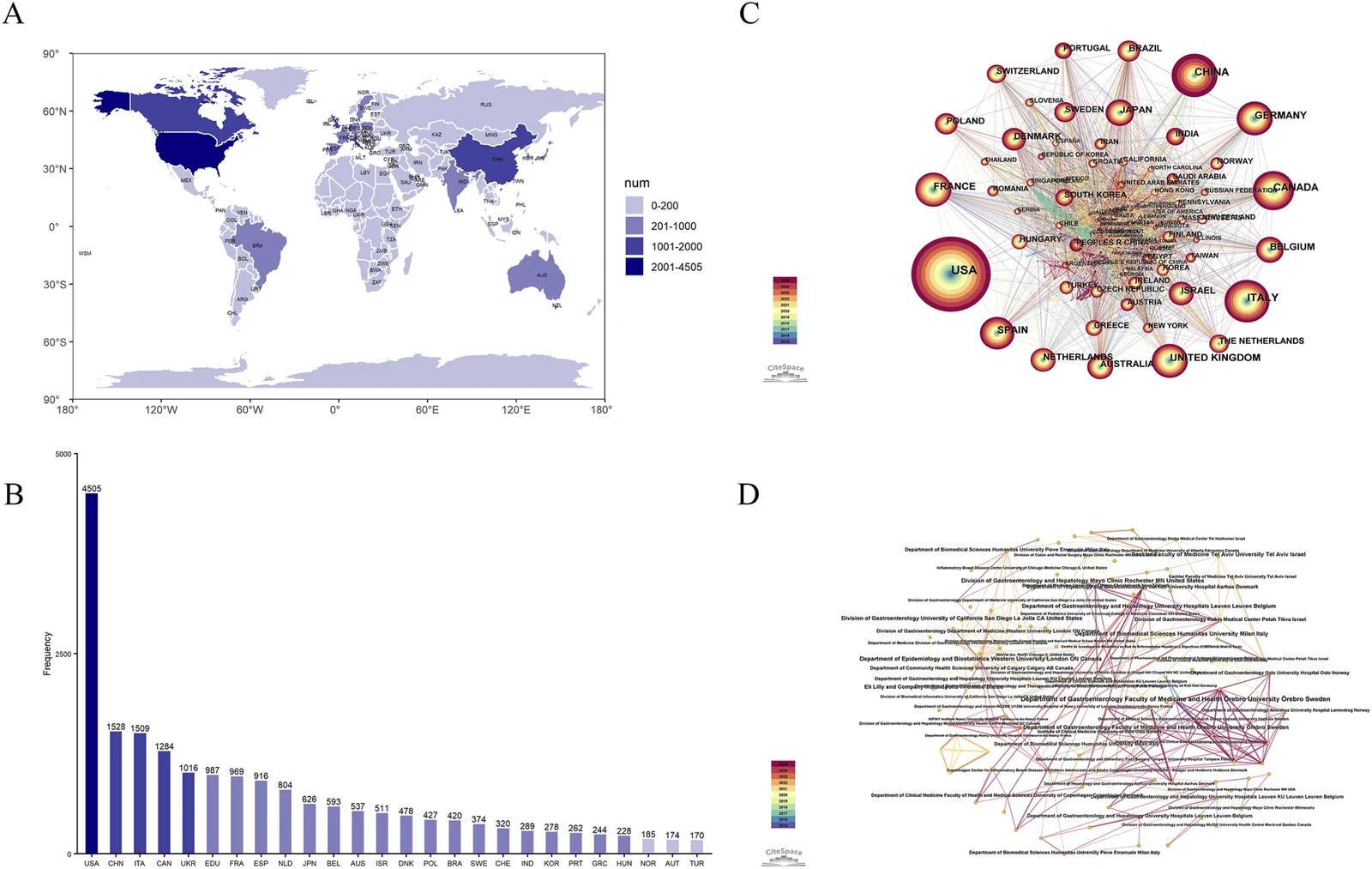
Country and institutional collaboration networks. **(A)** Geographic distribution of countries by publication volume. **(B)** Quantitative distribution of publications by country. **(C)** International collaboration network among countries. **(D)** Institutional collaboration network.

### 3.5 Institutional collaboration network

A total of 268 distinct institutions were identified as having contributed to the literature on biologic therapy for CD from 2015 to 2025. The institutional collaboration network comprised 517 co-authorship links, with each link representing at least one collaborative publication between two institutions (Figure 2D). The top 10 most productive institutions and their publication counts are presented in Table 2.

**TABLE 2:** Top 10 most productive institutions.

Rank	Institution	Country	Publications
1	Faculty of medicine and health Örebro University Örebro	Sweden	68
2	Western University London	United Kingdom	32
3	Mayo clinic	United States	31
4	Sackler Faculty of medicine Tel Aviv University	Israel	31
5	Humanitas University Milan	Italy	29
6	Harvard Medical School Boston	United States	25
7	Eli Lilly and Company Indianapolis	United States	24
8	University Hospitals Leuven Leuven	Belgium	23
9	University of California San Diego La Jolla	United States	23
10	Rabin Medical center Petah	Israel	22

### 3.6 Keyword Co-occurrence

Keyword co-occurrence analysis was performed using CiteSpace based on the keywords extracted from the merged database, yielding a network comprising 832 nodes and 4,716 links (Figure 3A). The keyword data were then imported into VOSviewer to generate a density visualization map (Figure 3D), and into RStudio using the ggplot2 and ggwordcloud packages to produce a word cloud (Figure 3E). The top 10 most frequent keywords were Crohn’s disease (n = 11,007), Inflammatory bowel disease (n = 9,522), Ulcerative colitis (n = 5,913), Middle aged (n = 3,696), Biological marker (n = 2,844), Biological product (n = 2,834), Treatment outcome (n = 2,240), Biological therapy (n = 2,003), C reactive protein (n = 1,623), and Tumor necrosis factor (n = 1,245). A centrality value greater than 0.1 indicates that a node serves as an important bridge within the network (Table 3; Figures 3A,D,E). It should be noted that the keyword analysis combined both author-supplied keywords and database-indexed terms. This hybrid approach ensures comprehensive coverage but may introduce synonym redundancy. To mitigate this, we retained both types for frequency analysis but applied semantic merging during cluster analysis using CiteSpace’s latent semantic indexing algorithm.

**FIGURE 3:**
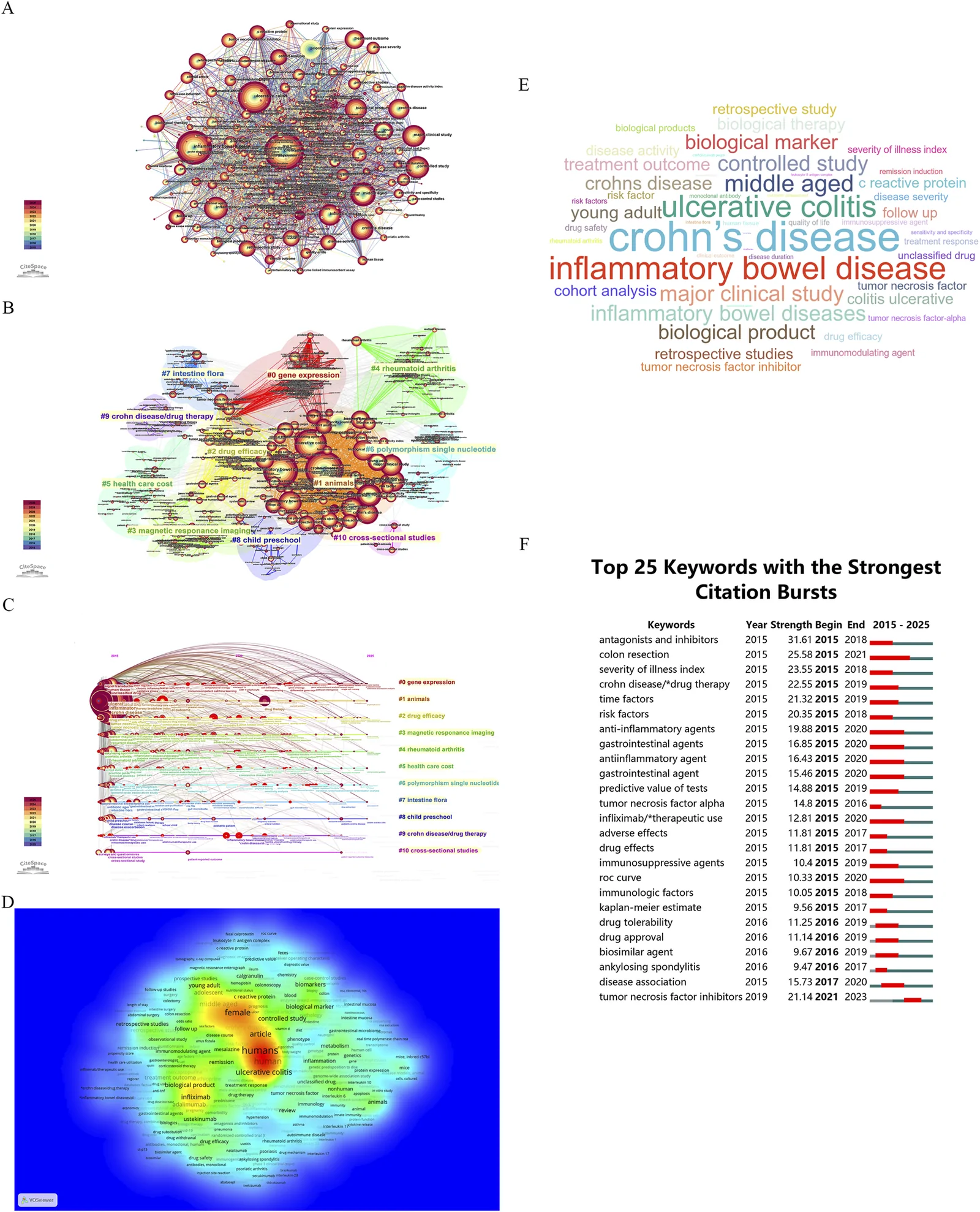
Keyword co-occurrence, clustering, word cloud, timeline, and citation burst analysis. **(A)** Keyword co-occurrence network. **(B)** Keyword cluster analysis. **(C)** Timeline visualization of keyword clusters. **(D)** Density visualization map of keywords. **(E)** Word cloud of high-frequency keywords. **(F)** Top keywords with the strongest citation bursts.

**TABLE 3:** Top 10 keywords.

Rank	Keyword	Frequency	Centrality
1	Crohn’s disease	11,007	1
2	Inflammatory bowel disease	9522	0.11
3	Ulcerative colitis	5913	0.15
4	Middle aged	3696	0.05
5	Biological marker	2844	0.35
6	Biological product	2834	0.25
7	Treatment outcome	2240	0.28
8	Biological therapy	2003	0.19
9	C Reactive protein	1623	0.17
10	Tumor necrosis factor	1245	0.24

### 3.7 Keyword clustering

To further characterize the structural organization of the research topics, a keyword-based cluster analysis was conducted, which identified 11 distinct and representative clusters, labeled as follows: #0 gene expression, #1 animals, #2 drug efficacy, #3 magnetic resonance imaging, #4 rheumatoid arthritis, #5 healthcare cost, #6 single nucleotide polymorphism, #7 intestinal flora, #8 preschool child, #9 Crohn’s disease/drug therapy, and #10 cross-sectional studies. The modularity Q value was 0.5991, exceeding the conventional threshold of 0.3, indicating that the cluster structure was statistically significant. The mean silhouette value was 0.8053 (>0.7), confirming that the clusters were internally homogeneous and well-defined (Figure 3B).

### 3.8 Keyword timeline chart

The keyword timeline visualization illustrates the temporal dynamics of different research themes over the study period. The horizontal axis represents the chronological sequence, while the vertical axis displays the 11 identified thematic clusters. In this visualization, node size corresponds to keyword frequency, the position of each node indicates the year in which the keyword first appeared, and the curvature of the connecting lines reflects the co-occurrence relationships among keywords across different time intervals (Figure 3C).

### 3.9 Keywords with citation bursts

To detect emergent research topics, we performed a citation burst analysis of the keywords, which identified 25 keywords exhibiting the strongest citation bursts (Figure 3F). Keywords citation burst analysis further elucidated the dynamic evolution of research foci over the study period. The intensive bursts of keywords represented by “antagonists and inhibitors” and “colon resection” reflected that early-stage research was primarily concentrated on therapeutic efficacy validation of biologic agents and surgical outcome management. Subsequently, the emergence of bursts such as “disease association” and “ROC curve” signaled a shift in research emphasis toward precision medicine and predictive modeling. This temporal transition illustrates a progressive maturation of the field, from initial efficacy confirmation to biomarker-driven patient stratification and outcome prediction. The temporal distribution of these bursts reflects the dynamic evolution of research foci in biologic therapy for CD over the study period.

## 4 Discussion

Crohn’s disease (CD) is a chronic, transmural inflammatory disorder with a predilection for the terminal ileum and adjacent colon, frequently complicated by strictures, fistulas, and perforation (Dolinger et al., 2024). Approximately 40%–50% of patients require surgical intervention within 10 years of diagnosis, and over half experience postoperative recurrence within the same period (Burisch et al., 2019). The pathogenesis of CD is multifactorial, involving genetic susceptibility (notably NOD2/CARD15, ATG16L1, and IRGM), immune dysregulation, gut microbiota alterations, and environmental factors. In CD patients, impaired innate immunity, thinning of the mucus layer, and increased epithelial permeability facilitate bacterial translocation and activation of immune cells, triggering excessive pro-inflammatory cytokine release. Concurrently, overactivation of adaptive immunity accelerates chronic intestinal inflammation (Saez et al., 2023). Gut microbiota dysbiosis is further characterized by reduced microbial diversity, enrichment of pathogenic bacteria, and compromised intestinal barrier function (Feng et al., 2023).

Conventional pharmacotherapy for CD primarily includes aminosalicylates, corticosteroids, immunomodulators, and enteral nutritional therapy. Among these, aminosalicylates, such as sulfasalazine, mesalazine, and balsalazide, exert their therapeutic effects by inhibiting cyclooxygenase activity and reducing prostaglandin synthesis, thereby suppressing the aggregation and adhesion of inflammatory cells and alleviating intestinal inflammation. This class of agents is characterized by a favorable safety profile and is particularly suitable for patients with mild-to-moderate CD and for maintenance therapy in remission phase (Hanauer and Strömberg, 2004). Corticosteroids, including methylprednisolone, prednisone, and budesonide, act by inhibiting arachidonic acid metabolism, reducing the production of inflammatory mediators, stabilizing lysosomal membranes, preventing the release of lytic enzymes from inflammatory cells, and suppressing the activation and proliferation of immune cells, ultimately attenuating the immune response. These agents offer the advantage of rapid onset of action; however, they are not recommended for long-term maintenance therapy (Sieber et al., 2022). Among immunomodulators, azathioprine and 6-mercaptopurine interfere with immune responses by inhibiting purine synthesis. They are indicated for patients who are steroid-dependent, intolerant to steroids, or have fistulizing disease. These agents have a slow onset of action and require regular monitoring of complete blood counts and liver function tests (Stournaras et al., 2021). Enteral nutritional therapy is also an important component of CD management, particularly in patients with mild-to-moderate active disease, in pediatric populations, and in the perioperative setting, where it offers distinct advantages.

Biologics are macromolecular drugs, including monoclonal antibodies and antibody-fusion proteins, engineered through modern biotechnology to target specific molecules involved in the inflammatory cascade of CD. These agents exert precise actions on their designated targets and have assumed a pivotal role in the management of CD, having become mainstay therapies for moderate-to-severe disease. Currently available biologics for CD include TNF-α inhibitors, integrin receptor antagonists, interleukin inhibitors, and other novel biologic agents.

TNF-α is a key pro-inflammatory cytokine that plays a critical role in the pathogenesis of CD. The most widely used biologics for inflammatory bowel disease, including infliximab, adalimumab, and golimumab, are TNF-α inhibitors. They exert their therapeutic effects by binding specifically to TNF-α, thereby blocking its interaction with cell surface receptors and attenuating the downstream inflammatory response. TNF-α inhibitors have demonstrated substantial efficacy in patients with moderate-to-severe CD (Regueiro et al., 2016). However, their use is associated with notable adverse effects, including infections (e.g., tuberculosis), infusion-related reactions, and local injection site reactions; long-term use has also been linked to an increased risk of lymphoma. Beyond these well-recognized adverse events, clinicians should remain vigilant for immune-related endocrinopathies, including thyroid dysfunction and adrenal insufficiency, which have been increasingly reported in patients receiving long-term monoclonal antibody therapy, particularly with anti-TNF-α agents. Although these endocrine disturbances occur less frequently than classical infusion reactions or infectious complications, they may profoundly affect metabolic homeostasis, energy balance, and overall quality of life, warranting routine endocrine surveillance—including thyroid function tests and morning cortisol measurements—in high-risk populations or patients presenting with non-specific symptoms such as fatigue, weight changes, or mood disturbances.

Infliximab, the first FDA-approved biologic for CD, is a chimeric human-murine monoclonal IgG1 antibody that neutralizes soluble and membrane-bound TNF-α, induces T-cell apoptosis, and exerts antibody-dependent cell-mediated cytotoxicity. It demonstrates robust efficacy in inducing and maintaining remission in moderate-to-severe CD, particularly in fistulizing disease and patients with inadequate response to conventional therapy, with notable benefits in mucosal healing (Akobeng and Zachos, 2004). The standard regimen is 5 mg/kg intravenously at weeks 0, 2, and 6, followed by maintenance every 8 weeks (Cornillie et al., 2001). However, long-term use is complicated by anti-infliximab antibodies (ATI), which are associated with a twofold higher risk of infusion reactions. In addition, infliximab increases the risk of infections, including upper respiratory, urinary tract, and opportunistic infections. Pretreatment screening for tuberculosis (tuberculin skin testing and chest radiography) is mandatory, and close monitoring throughout therapy is essential. Rare but serious adverse events, including lymphoma, sepsis, and death, have also been reported (Siegel et al., 2006).

Adalimumab, a recombinant human IgG1 monoclonal antibody against TNF-α, offers convenient subcutaneous administration and has demonstrated favorable efficacy in infliximab-nonresponsive or -intolerant patients, improving their quality of life (Ho et al., 2008). It shows particularly robust efficacy in anti-TNF-naïve patients with moderate-to-severe active CD, and is associated with lower costs and reduced hospitalization rates compared with infliximab (Yu et al., 2009). Certolizumab pegol, a PEGylated Fab’ fragment of a humanized anti-TNF-α antibody approved by the FDA in 2008, has shown efficacy in moderate-to-severe CD, especially in patients with elevated CRP, though its long-term safety (serious infections and lymphoma) requires further investigation (Ho et al., 2008). Natalizumab, a recombinant humanized IgG4 monoclonal antibody against α4 integrin that blocks α4β1 and α4β7 heterodimers, inhibits leukocyte trafficking into intestinal mucosa. It is effective in inducing and maintaining remission in moderate-to-severe CD, but its use is limited by the risk of progressive multifocal leukoencephalopathy (PML), necessitating careful risk-benefit assessment (Ko and Bressler, 2007).

Among integrin inhibitors, vedolizumab—the only gut-selective agent approved for CD—binds to α4β7 integrin on lymphocytes, blocking MAdCAM-1 interaction and preventing lymphocyte migration into intestinal inflammatory sites, thereby minimizing systemic immunosuppression (Bach and Taleban, 2020). It has demonstrated efficacy in inducing and maintaining remission, promoting mucosal healing, and sustaining long-term benefits in moderate-to-severe CD, particularly in patients with inadequate response or intolerance to TNF-α inhibitors (Sands et al., 2017). Risankizumab, an IL-23 inhibitor, received regulatory approval for CD in 2022 (FDA) and 2023 (EMA), with recent 2024 guideline updates from ECCO and 2025 ACG positioning it as a first-line biologic option for moderate-to-severe CD, particularly in anti-TNF-naïve patients. Two phase III trials have confirmed its superiority in inducing symptomatic remission and maintaining efficacy (D'Haens et al., 2022; Ferrante et al., 2022), with endoscopic endpoints aligned with STRIDE-II treat-to-target strategies (Turner et al., 2021). Ustekinumab, targeting the shared p40 subunit of IL-12/IL-23, blocks Th1 and Th17 differentiation and proliferation, demonstrating robust efficacy in inducing and maintaining remission in moderate-to-severe CD (Johnson et al., 2023), including as salvage therapy in TNF-α inhibitor-refractory patients (Feagan et al., 2016). A meta-analysis confirmed that IL-23-targeting biologics are effective and safe for inducing and maintaining clinical and endoscopic remission (25). Ch5D12, a chimeric anti-CD40 monoclonal antibody blocking the CD40/CD40L co-stimulatory pathway, has shown good tolerability and a 72% clinical response rate in moderate-to-severe CD, serving as a potential induction option (Kasran et al., 2005). Other agents under investigation include anti-IL-6 receptor mAbs, which inhibit vascular adhesion molecules and induce lamina propria cell apoptosis, and ABT-874 (anti-IL-12 mAb), which reduces IL-12, IFN-γ, and TNF-α secretion and sustains remission (Mannon et al., 2004). Fontolizumab, a humanized anti-IFN-γ antibody, directly neutralizes IFN-γ with good tolerability and low immunogenicity (Reinisch et al., 2006).

Among other monoclonal antibodies, secukinumab—a fully human selective IL-17A monoclonal antibody—has been investigated in CD. IL-17 is a key pro-inflammatory cytokine secreted by Th17 cells and plays an important role in intestinal inflammation. However, early validation studies of secukinumab in moderate-to-severe CD did not meet the expected therapeutic efficacy. Therefore, the role of IL-17-targeting agents in CD warrants further investigation (Hueber et al., 2012). Granulocyte-macrophage colony-stimulating factor (GM-CSF) is involved in the recruitment and activation of inflammatory cells. Biologic agents targeting GM-CSF have shown anti-inflammatory effects in animal models and preliminary human studies, offering novel insights into the treatment of CD, although further research is needed (Roth et al., 2012). Additionally, novel oral anti-TNF-α agents, such as OPRX-106 and V565, are currently under development. In terms of clinical efficacy, infliximab has demonstrated induction of clinical remission in approximately 60% of patients with moderate-to-severe CD, with endoscopic remission rates ranging from 30% to 40% at 1 year, as defined by a Crohn’s Disease Endoscopic Index of Severity (CDEIS) score <3 or a Simple Endoscopic Score for Crohn’s Disease (SES-CD) < 3. Similarly, vedolizumab and ustekinumab have shown comparable efficacy in achieving clinical remission, yet their safety profiles differ significantly, particularly regarding systemic immunosuppression.

The advent of biologic agents has significantly improved clinical response and remission rates in patients with active Crohn’s disease, and has made mucosal healing a realistic therapeutic goal. In certain clinical scenarios, combination therapy with biologics and other agents has demonstrated superior efficacy compared with biologic monotherapy (Colombel et al., 2010). Conventionally, a step-up approach has been recommended for the treatment of CD. However, increasing attention has been directed toward the top-down strategy in recent years. Evidence suggests that early adoption of a top-down regimen in newly diagnosed patients may achieve better clinical outcomes than conventional step-up therapy. Nevertheless, the use of biologics is associated with challenges, including secondary loss of response, central demyelinating disorders, and drug-induced lupus-like syndrome. Early biologic intervention has been shown to significantly reduce the eventual need for surgery in CD patients. Therefore, a rigorous risk-benefit assessment is essential to guide treatment decisions and to formulate optimal early intervention strategies (Law et al., 2024).

Our bibliometric analysis revealed that research on biologic therapy for CD has remained highly active from 2015 to 2025, with annual publication output showing a consistent upward trend, reflecting the growing global interest in this field. A total of 15,828 publications were ultimately included, and the substantial volume of literature underscores the sustained research efforts in this area. In terms of geographic distribution, the United States, located in the Western and Northern Hemispheres, contributed the largest number of publications, followed by China, Italy, Canada, and the United Kingdom, all situated in the Eastern and/or Northern Hemispheres. The global epidemiology of CD is markedly uneven, with the highest prevalence observed in traditionally developed countries, whereas the most rapid increase in incidence is occurring in emerging economies—a pattern that may be closely associated with globalization-driven changes in dietary habits, lifestyle, and daily routines.

High-frequency keyword analysis of research on biologic therapy for Crohn’s disease over the study period clearly delineated a complete academic logic chain extending from “disease definition” to “clinical outcomes.” The research framework was anchored by “Crohn’s disease” (n = 11,007) as the absolute core, linking upward to the broader disciplinary framework of “Inflammatory bowel disease” (n = 9,522) and maintaining sustained comparative alignment with “Ulcerative colitis” (n = 5,913) at the lower level. From a bibliometric perspective, this stable tri-level structure suggests that CD research remains firmly embedded within the broader IBD context. The high co-occurrence frequencies may reflect sustained efforts to delineate CD-specific mechanisms through systematic comparisons with UC, although such inferences derived from keyword frequencies should be interpreted with caution and complemented by manual content verification. The combined frequency of these three terms exceeded 26,000 occurrences, constituting the predominant share of all keyword frequencies and suggesting that the research foundation of this field remains highly concentrated within the framework of disease taxonomy. The near-identical frequencies of “Biological marker” (n = 2,844) and “Biological product” (n = 2,834) formed a highly coupled research dyad. This observation is noteworthy from a bibliometric standpoint, as it may indicate that biomarker-related research has become increasingly integrated with biologic clinical trials and real-world studies. However, it is important to emphasize that keyword frequency reflects research attention rather than clinical adoption or therapeutic value. Cautiously interpreted, this coupled pattern could suggest a gradual shift in research emphasis—from a primary focus on efficacy validation toward greater attention to patient stratification, response prediction, and treatment personalization. Nevertheless, describing this as a ‘complete paradigm shift’ based solely on keyword frequencies would be overinterpretation; the trend is more appropriately characterized as an evolving research emphasis rather than a confirmed clinical transformation. Notably, “Biological therapy” (n = 2,003), as a broader concept of treatment strategy, appeared with significantly lower frequency than “Biological product,” implying that current research remains predominantly product-centric, while optimization of therapeutic strategies remains relatively underexplored and may constitute an important growth area for future investigation. The therapeutic evaluation system exhibited a dual-core pattern extending from “drug efficacy” to “treatment outcomes.” “Treatment outcome” (n = 2,240) emerged as the core component of clinical value assessment, with “outcome” pointing toward comprehensive clinical benefits in the real-world setting—including remission rates, mucosal healing, quality of life, surgical risk, and long-term prognosis. This pattern suggests that the research perspective in the field is transitioning from “whether the drug works” to “whether the drug leads to meaningful clinical outcome improvements,” reflecting a profound shift from pharmacodynamic-oriented thinking to patient-centered outcome evaluation. Classic therapeutic targets and clinical biomarkers jointly constitute the two pillars of precision medicine. “C reactive protein” (n = 1,623) and “Tumor necrosis factor” (n = 1,245) represent two parallel pathways within precision medicine. CRP, as the most widely used clinical biomarker for monitoring inflammatory activity, achieved high frequency, which may reflect the dual emphasis in the literature on both disease activity monitoring (CRP) and mechanistic target investigation (TNF). However, it should be noted that the higher frequency of CRP over TNF does not necessarily indicate greater clinical importance; rather, it may simply reflect the broader applicability of CRP measurement across diverse study designs and patient populations. Meanwhile, TNF, as the first successfully targeted therapeutic target, not only established the historical benchmark status of anti-TNF-α therapy but also serves as the reference standard for comparative efficacy evaluation of all subsequent biologics and next-generation small-molecule agents. The higher frequency of CRP over TNF reflects a noteworthy clinical reality: in routine clinical practice, the need for dynamic monitoring of disease activity may be more urgent and more widely applicable than the need to explore mechanistic targets. However, it should be noted that although CRP remains the most widely accessible biomarker for acute-phase inflammation, fecal calprotectin (FC) has emerged as a more sensitive and gut-specific non-invasive marker for intestinal mucosal inflammation in CD. Accumulating evidence indicates that FC correlates more closely with endoscopic disease activity than serum CRP, particularly in patients with isolated ileal involvement or those with a normal systemic inflammatory response. The dynamic monitoring of FC offers practical advantages in routine follow-up, enabling timely therapeutic adjustments without repeated endoscopic assessments, and its integration into treat-to-target strategies has been strongly recommended by recent guidelines. Thee age-orientation of the research population remains pronounced, highlighting an evidence gap in special populations that warrants urgent attention. The presence of “Middle aged” as a demographic keyword among the high-frequency terms may indicate that a substantial proportion of published research has focused on middle-aged and older populations. This focus aligns with the age distribution pattern of CD onset (the second peak, 50–70 years) and reflects the field’s emphasis on long-term disease management, complication prevention, and malignancy surveillance—issues particularly relevant to aging populations. However, evidence accumulation for special populations—including adolescents (the first peak of onset, 15–30 years) and pregnant women—remains markedly insufficient. This research gap not only limits the breadth of coverage of clinical guidelines but also constitutes an important growth direction for future investigation. Citation burst analysis further elucidated the dynamic evolution of research foci over the study period. Between 2015 and 2018, intensive bursts of keywords represented by “antagonists and inhibitors” and “colon resection” reflected that early-stage research was predominantly concentrated on efficacy validation of biologics and surgical outcome management. Between 2017 and 2020, the emergence of bursts such as “disease association” and “ROC curve” signaled a shift in research emphasis toward precision medicine and predictive modeling; concurrently, the bursts of “biosimilar agent” and “drug approval” reflected the real-time impact of biosimilar market entry and regulatory review on research hotspots in this field. Notably, the resurgence of “tumor necrosis factor inhibitors” between 2021 and 2023 indicates that the research logic of the field has evolved from the exploratory phase of introducing novel agents to a comparative optimization phase centered on how to select and sequence among multiple available therapies. This three-phase evolutionary pattern corroborates the natural disciplinary progression of CD biologic therapy research—from new drug introduction to therapeutic strategy optimization.

Beyond the bibliometric trends, the clinical impact of this research evolution is best reflected in the changing epidemiology of CD-related complications. Over the past years, the widespread adoption of biologic agents has been associated with a marked reduction in the need for intestinal resection. Observational data from large population-based cohorts indicate that the cumulative 5-year surgical resection rate has declined from approximately 40% in the pre-biologic era to less than 25% in recent years, with a concurrent 30% reduction in postoperative endoscopic recurrence rates. This epidemiological shift not only validates the real-world effectiveness of biologics but also underscores that the paradigm has evolved beyond mere confirmation of whether biologics work to a more nuanced optimization of when, in whom, and in what sequence they work best.

Several limitations of this study should be acknowledged. The English-language restriction, database coverage heterogeneity, and potential author/institution name variations may have introduced selection and classification biases. Additionally, keyword-based and cluster analyses are inherently dependent on indexing accuracy, synonym standardization, and algorithm parameters, with citation bursts reflecting frequency surges rather than underlying causes. Finally, bibliometric indicators reflect research activity and publication trends rather than direct clinical evidence, and should therefore be interpreted with caution.

## 5 Conclusion

From 2015 to 2025, research on biologic therapy for Crohn’s disease has demonstrated consistent growth and thematic evolution, characterized by biologics as the central therapeutic intervention, controlled clinical studies as the primary methodological approach, biomarkers as an increasingly important tool for precision medicine, and treatment outcomes as the key measure of clinical value. The maturation of this ecosystem not only suggests a progressive transition in the management of Crohn’s disease—from adjunctive therapy toward increasingly stratified and biomarker-informed approaches—but also may offer insights for biologic research in other immune-mediated inflammatory diseases, though cross-disease generalizability requires separate investigation. Our bibliometric findings indicate a progressive research emphasis—from initial efficacy validation toward biomarker-driven stratification and comparative effectiveness evaluation. Future research directions may include individualized sequencing strategies for biologics, head-to-head comparisons between novel small-molecule agents and conventional biologics, and the generation of efficacy and safety evidence in understudied populations beyond the middle-aged cohort, including adolescents and pregnant women. The key priority areas emerging from the data include: (1) head-to-head comparative studies among biologics and between biologics and novel small-molecule agents; (2) biomarker-driven patient stratification and predictive modeling; and (3) expanded research in understudied populations, particularly pediatric and pregnant cohorts. Progress in these areas is expected to further bridge the gap between clinical trial evidence and real-world clinical decision-making, ultimately advancing the field toward the overarching goal of transitioning from population-average efficacy to individually optimized therapeutic outcomes.

## Data Availability

The original contributions presented in the study are included in the article/supplementary material, further inquiries can be directed to the corresponding author.

## References

[B1] Akobeng A. K. Zachos M. (2004). Tumor necrosis factor-alpha antibody for induction of remission in Crohn's disease. Cochrane Database Syst. Rev. 2003 (4), CD003574. 10.1002/14651858 14974022 PMC8721561

[B2] Bach L. E. Taleban S. (2020). Vedolizumab (VDZ) for UC and CD: still safe and effective after all these years. Dig. Dis. Sci. 65, 1882–1884. 10.1007/s10620-020-06212-7 32212020

[B3] Burisch J. Kiudelis G. Kupcinskas L. Kievit H. A. L. Andersen K. W. Andersen V. (2019). Natural disease course of Crohn's disease during the first 5 years after diagnosis in a European population-based inception cohort: an Epi-IBD study. Gut 68, 423–433. 10.1136/gutjnl-2017-315568 29363534

[B4] Colombel J. F. Sandborn W. J. Reinisch W. Mantzaris G. J. Kornbluth A. Rachmilewitz D. (2010). Infliximab, azathioprine, or combination therapy for Crohn's disease. N. Engl. J. Med. 362 (362), 1383–1395. 10.1056/NEJMoa0904492 20393175

[B5] Cornillie F. Shealy D. D'Haens G. Geboes K. Van Assche G. Ceuppens J. (2001). Infliximab induces potent anti-inflammatory and local immunomodulatory activity but no systemic immune suppression in patients with Crohn's disease. Aliment. Pharmacol. Ther. 15, 463–473. 10.1046/j.1365-2036.2001.00956.x 11284774

[B6] D'Haens G. Panaccione R. Baert F. Bossuyt P. Colombel J. F. Danese S. (2022). Risankizumab as induction therapy for Crohn's disease: results from the phase 3 ADVANCE and MOTIVATE induction trials. Lancet. May 28 (399), 2015–2030. 10.1016/S0140-6736(22)00467-6 35644154

[B7] Dolinger M. Torres J. Vermeire S. (2024). Crohn's disease. Lancet 403 (403), 1177–1191. 10.1016/S0140-6736(23)02586-2 38437854

[B8] Feagan B. G. Sandborn W. J. Gasink C. Jacobstein D. Lang Y. Friedman J. R. (2016). Ustekinumab as induction and maintenance therapy for Crohn's Disease. N. Engl. J. Med. 17 (375), 1946–1960. 10.1056/NEJMoa1602773 27959607

[B9] Feng R. Tian Z. Mao R. Ma R. Luo W. Zhao M. (2023). Gut microbiome-generated phenylacetylglutamine from dietary protein is associated with Crohn's Disease and exacerbates Colitis in mouse model possibly *via* platelet activation. J. Crohns Colitis 17, 1833–1846. 10.1093/ecco-jcc/jjad098 37350766

[B10] Ferrante M. Panaccione R. Baert F. Bossuyt P. Colombel J. F. Danese S. (2022). Risankizumab as maintenance therapy for moderately to severely active Crohn's disease: results from the multicentre, randomised, double-blind, placebo-controlled, withdrawal phase 3 FORTIFY maintenance trial. Lancet. May 28 (399), 2031–2046. 10.1016/S0140-6736(22)00466-4 35644155

[B11] Hanauer S. B. Strömberg U. (2004). Oral Pentasa in the treatment of active Crohn's disease: a meta-analysis of double-blind, placebo-controlled trials. Clin. Gastroenterol. Hepatol. 2, 379–388. 10.1016/s1542-3565(04)00122-3 15118975

[B12] Ho G. T. Smith L. Aitken S. Lee H. M. Ting T. Fennell J. (2008). The use of adalimumab in the management of refractory Crohn's disease. Aliment. Pharmacol. Ther. 27 (27), 308–315. 10.1111/j.1365-2036.2007.03583.x 18081730

[B13] Hueber W. Sands B. E. Lewitzky S. Vandemeulebroecke M. Reinisch W. Higgins P. D. (2012). Secukinumab, a human anti-IL-17A monoclonal antibody, for moderate to severe Crohn's disease: unexpected results of a randomised, double-blind placebo-controlled trial. Gut 61, 1693–1700. 10.1136/gutjnl-2011-301668 22595313 PMC4902107

[B14] Johnson A. M. Barsky M. Ahmed W. Zullow S. Galati J. Jairath V. (2023). The real-world effectiveness and safety of ustekinumab in the treatment of Crohn's Disease: results from the SUCCESS consortium. Am. J. Gastroenterol. 118, 317–328. 10.14309/ajg.0000000000002047 36191274

[B15] Kasran A. Boon L. Wortel C. H. Hogezand R. A. Schreiber S. Goldin E. (2005). Safety and tolerability of antagonist anti-human CD40 Mab ch5D12 in patients with moderate to severe Crohn's disease. Aliment. Pharmacol. Ther. 22, 111–122. 10.1111/j.1365-2036.2005.02526.x 16011669

[B16] Ko H. H. Bressler B. (2007). Natalizumab: pharmacology, clinical efficacy and safety in the treatment of patients with Crohn's disease. Expert Rev. Gastroenterol. Hepatol. 1, 29–39. 10.1586/17474124.1.1.29 19072431

[B17] Law C. C. Y. Tkachuk B. Lieto S. Narula N. Walsh S. Colombel J. F. (2024). Early biologic treatment decreases risk of surgery in Crohn's Disease but not in ulcerative colitis: systematic review and meta-analysis. Inflamm. Bowel Dis. 30, 1080–1086. 10.1093/ibd/izad149 37506265 PMC11219475

[B18] MacDonald J. K. Nguyen T. M. Khanna R. Timmer A. (2016). Anti-IL-12/23p40 antibodies for induction of remission in Crohn's disease. Cochrane Database Syst. Rev. Nov. 25, Cd007572. 10.1002/14651858.CD007572.pub3 27885650 PMC6464484

[B19] Mannon P. J. Fuss I. J. Mayer L. Elson C. O. Sandborn W. J. Present D. (2004). Anti-interleukin-12 antibody for active Crohn's disease. N. Engl. J. Med. 351, 2069–2079. 10.1056/NEJMoa033402 15537905

[B20] Nielsen O. H. Ainsworth M. A. (2013). Tumor necrosis factor inhibitors for inflammatory bowel disease. N. Engl. J. Med. 369, 754–762. 10.1056/NEJMct1209614 23964937

[B21] Regueiro M. Feagan B. G. Zou B. Johanns J. Blank M. A. Chevrier M. (2016). Infliximab reduces endoscopic, but not clinical, recurrence of Crohn's Disease after ileocolonic resection. Gastroenterology 150, 1568–1578. 10.1053/j.gastro.2016.02.072 26946343

[B22] Reinisch W. Hommes D. W. Van Assche G. Colombel J. F. Gendre J. P. Oldenburg B. (2006). A dose escalating, placebo controlled, double blind, single dose and multidose, safety and tolerability study of fontolizumab, a humanised anti-interferon gamma antibody, in patients with moderate to severe Crohn's disease. Gut 55, 1138–1144. 10.1136/gut.2005.079434 16492717 PMC1856289

[B23] Roth L. MacDonald J. K. McDonald J. W. Chande N. (2012). Sargramostim (GM-CSF) for induction of remission in Crohn's disease: a cochrane inflammatory bowel disease and functional bowel disorders systematic review of randomized trials. Inflamm. Bowel Dis. 18, 1333–1339. 10.1002/ibd.22973 22552871

[B24] Saez A. Herrero-Fernandez B. Gomez-Bris R. Sánchez-Martinez H. Gonzalez-Granado J. M. (2023). Pathophysiology of inflammatory bowel disease: innate immune System. Int. J. Mol. Sci. Jan. 12, 24. 10.3390/ijms24021526 36675038 PMC9863490

[B25] Sandborn W. J. Feagan B. G. Rutgeerts P. Hanauer S. Colombel J. F. Sands B. E. (2013). Vedolizumab as induction and maintenance therapy for Crohn's disease. N. Engl. J. Med. 369 (369), 711–721. 10.1056/NEJMoa1215739 23964933

[B26] Sands B. E. Sandborn W. J. Van Assche G. Lukas M. Xu J. James A. (2017). Vedolizumab as induction and maintenance therapy for Crohn's Disease in patients Naïve to or who have failed tumor necrosis factor antagonist therapy. Inflamm. Bowel Dis. 23, 97–106. 10.1097/MIB.0000000000000979 27930408

[B27] Sieber A. Aberra F. N. Bonhomme B. McKeever L. Lewis J. D. (2022). Influence of corticosteroid use on Short- and long-term outcomes of biologic therapy for inflammatory bowel diseases. Dig. Dis. Sci. 67, 5168–5176. 10.1007/s10620-021-07361-z 35094254

[B28] Siegel C. A. Hur C. Korzenik J. R. Gazelle G. S. Sands B. E. (2006). Risks and benefits of infliximab for the treatment of Crohn's disease. Clin. Gastroenterol. Hepatol. 4, 1017–1024; quiz 1976. 10.1016/j.cgh.2006.05.020 16843733

[B29] Stournaras E. Qian W. Pappas A. Hong Y. Y. Shawky R. Raine T. (2021). Thiopurine monotherapy is effective in ulcerative colitis but significantly less so in Crohn's disease: long-term outcomes for 11 928 patients in the UK inflammatory bowel disease bioresource. Gut 70, 677–686. 10.1136/gutjnl-2019-320185 33004550 PMC7948184

[B30] Turner D. Ricciuto A. Lewis A. D'Amico F. Dhaliwal J. Griffiths A. M. (2021). STRIDE-II: an update on the selecting therapeutic targets in inflammatory bowel disease (STRIDE) initiative of the International Organization for the Study of IBD (IOIBD): determining therapeutic goals for treat-to-target strategies in IBD. Gastroenterology 160, 1570–1583. 10.1053/j.gastro.2020.12.031 33359090

[B31] Yu A. P. Johnson S. Wang S. T. Atanasov P. Tang J. Wu E. (2009). Cost utility of adalimumab *versus* infliximab maintenance therapies in the United States for moderately to severely active Crohn's disease. Pharmacoeconomics 27, 609–621. 10.2165/11312710-000000000-00000 19663531

